# Autologous induced pluripotent stem cell-derived four-organ-chip

**DOI:** 10.2144/fsoa-2019-0065

**Published:** 2019-09-10

**Authors:** Anja Patricia Ramme, Leopold Koenig, Tobias Hasenberg, Christine Schwenk, Corinna Magauer, Daniel Faust, Alexandra K Lorenz, Anna-Catharina Krebs, Christopher Drewell, Kerstin Schirrmann, Alexandra Vladetic, Grace-Chiaen Lin, Stephan Pabinger, Winfried Neuhaus, Frederic Bois, Roland Lauster, Uwe Marx, Eva-Maria Dehne

**Affiliations:** 1 TissUse GmbH, Oudenarder Str. 16, 13347 Berlin, Deutschland; 2 Technische Universität Berlin, Medizinische Biotechnologie, Gustav-Meyer-Allee 25, 13355 Berlin, Deutschland; 3 The University of Manchester, Physics of Fluids & Soft Matter Group, Oxford Road, Manchester M13 9PL, UK; 4 AIT Austrian Institute of Technology GmbH, Giefinggasse 4, 1210 Vienna, Austria; 5 INERIS, METO unit, Parc ALATA BP2, 60550 Verneuil en Halatte, France

**Keywords:** differentiation, four-organ-chip, induced pluripotent stem cells, microphysiological system, multi-organ-chip

## Abstract

Microphysiological systems play a pivotal role in progressing toward a global paradigm shift in drug development. Here, we designed a four-organ-chip interconnecting miniaturized human intestine, liver, brain and kidney equivalents. All four organ models were predifferentiated from induced pluripotent stem cells from the same healthy donor and integrated into the microphysiological system. The coculture of the four autologous tissue models in one common medium deprived of tissue specific growth factors was successful over 14-days. Although there were no added growth factors present in the coculture medium, the intestine, liver and neuronal model maintained defined marker expression. Only the renal model was overgrown by coexisting cells and did not further differentiate. This model platform will pave the way for autologous coculture cross-talk assays, disease induction and subsequent drug testing.

## Lay abstract

Models of four human organs – intestine, liver, brain, kidney – were cultivated in a micro-bioreactor, called microphysiological system. The organ models were placed in distinct compartments of the bioreactor which were connected by a microfluidic network. This fluidic circulation enabled a cross-talk between the four organ models similar to the human blood circulation connecting the organs in the human body. The organ models were engineered from induced pluripotent stem cells derived of a single donor. Therewith, all organ models shared the same genetic background. This might allow for a more accurate and patient specific testing of drugs in future.

## Graphical abstract

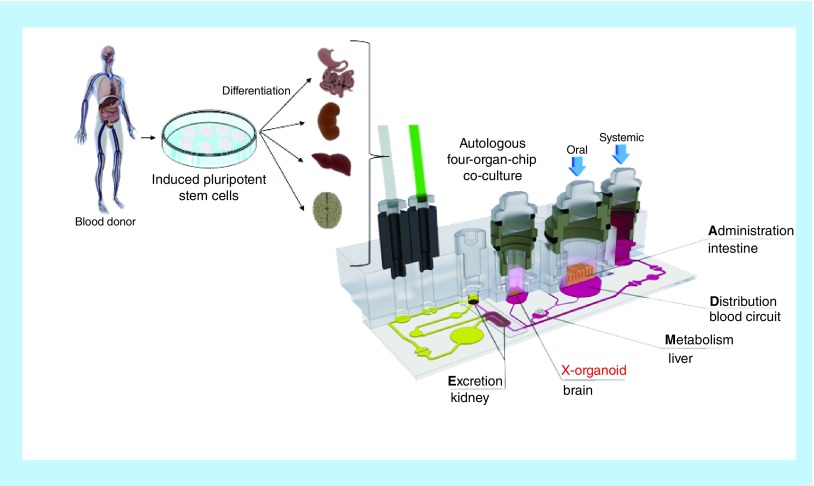

Microphysiological systems (MPS) have evolved greatly in sophistication during the past decade. Their increased physiological relevance enhances the translatability of assay readouts and results to the human situation [1]. Various systems mimicking *in vivo*-like tissue architecture and physiological flow conditions have already been shown to be highly valuable in the various stages of the drug development process [2]. So far, predominantly cell lines or primary tissues from different donors have been used for the cocultivation in MPS. However, a different genetic background of the tissues during the cocultivation is a major drawback when opting for complex multi-organ systems eventually even integrating the immune components. Therefore, MPS need to be established from tissues of one autologous source. Organ differentiation from a single induced pluripotent stem cell (iPSC) source could provide a solution to this challenge. A model recapitulating the effects of a treatment on iPSC-derived organoids from a healthy donor and, similarly, the generation of tailored disease models by using iPSC-derived organoids from a diseased donor or by genome editing on healthy iPSCs show great promise. MPSs are capable of emulating human biology *in vitro* at the smallest biologically acceptable scale. The dynamic fluid flow is adjusted to enable physiological nutrition and oxygen supply of the tissues mimicking organ function with a minimal use of human cells and tissues [3]. Multi-organ MPSs, furthermore, can mimic complex biological processes involving organ–organ interaction, system homeostasis and pharmacokinetics. These systems will enable quicker, more accurate, cost-effective and clinically relevant testing of drugs [3,4].

Many stem cell-based approaches for cell differentiation have emerged in recent years. Also, some studies combining stem cells and MPS have been published [5–10]. Prominent examples include: functional differentiation of iPSCs into cardiomyocytes- or hepatocyte-like cells-on-a-chip [11]; differentiation and cultivation of three-dimensional (3D) brain organoids in a perfusable organ-on-a-chip system [12]; and mesenchymal stem cell cultures and differentiation in microfluidic devices [5]. However, to the best of our knowledge, there is no multi-organ MPS coculturing solely iPSC-derived organ equivalents in a common media circuit.

Conventional iPSC-derived cultures require highly specialized culture conditions. Each cell type, for example, demands for its own set of adjusted growth factors. Therefore, the coculture of several organ equivalents is not straightforwardly possible using routine culture methods. To address this issue, we adapted our recently published microphysiological multi-organ-chip (MOC) platform to host the organ equivalents desired [13–15]. It has been shown previously that the MOC can coculture tissue models of various origins, based on cell lines or primary cells, for up to 28 days under homeostasis [13,14]. We assumed that pre-differentiated organ models for the intestine, liver, brain and kidney of iPSC origin could similarly maintain their phenotype under the culture conditions of the microfluidic device – even without the addition of tissue-specific growth factors. Here, we describe the coculture of four iPSC-derived organ equivalents from one single donor in a four-organ-chip. The intestine, liver and neuronal models maintained their phenotype during the 14 days of coculture in a common, growth factor-deprived medium. Only the renal model was overgrown by coexisting cells and did not further differentiate.

## Materials & methods

### Chip fabrication

Standard soft lithography and replica molding of polydimethylsiloxane (PDMS; Sylgard 184, Dow Corning, MI, USA) were applied for the fabrication of the chip (HUMIMIC Chip4, TissUse GmbH, Berlin, Germany). Briefly, two master molds were fabricated by aluminum milling – one mold for the surrogate blood circuit and one for the excretory circuit. An adapter plate (polycarbonate, 10 mm) containing access holes was treated with a silicon rubber additive (1200 OS Primer, Dow Corning) and clamped to the master mold for the excretory circuit. Screws were inserted into the holes of the adapter plate to generate the PDMS-free culture compartments and the PDMS membranes constituting the two on-chip micropumps. Liquid PDMS (10:1 v/v ratio of PDMS to curing agent) was injected into this casting station. The setup was incubated at 80°C for at least 60 min. The PDMS slice bonds fluid-tight to the adapter plate. A second PDMS slice of the bottom blood circuit was prepared without an adapter plate and without through holes. After curing, the PDMS slices were treated with low-pressure ambient air plasma (Pico, Diener GmbH, Ebhausen, Germany) and immersed in a 2% (3-Glycidyloxypropyl) trimethoxysilane aqueous solution (440167, Sigma-Aldrich, MO, USA) for 20 min at room temperature. In parallel, a precut cell culture ready-to-use polycarbonate membrane (pore size: 1.0 μm, pore density: 2 × 10^6^ cm^-2^, thickness: 24.0 μm, it4ip S.A., Louvain-la-Neuve, Belgium) was also treated with plasma and immersed in 1% (3-Aminopropyl) triethoxysilane aqueous solution (A3648, Sigma-Aldrich) for 20 min at room temperature. After drying, the membrane was placed on the space provided on the bottom PDMS slice exactly at the position of the glomerulus and tubules compartments. Both PDMS slices were aligned. Critical PDMS membranes were drawn upward using a vacuum of less than -20 kPa to prohibit their bonding. The chip was then incubated for at least 24 h at 80°C, which realized a fluid-tight bonding of the two PDMS slices. The chips were rinsed with ethanol, phosphate buffered saline (PBS) and, subsequently, cultivation medium. Thereafter, the chip was kept at 37°C in a humid incubator until the start of the experiment.

### Chip-based cocultures

The excretory circuits of each chip were loaded with 1 × 10^6^ iPSC-derived renal cells and after 6 days the coculture experiment was started. At day zero 14 chips were equipped with liver spheroids (total of 1 × 10^6^ iPSC-derived hepatocytes with iPSC-derived stromal cells), the intestine model (approx. 0.3 × 10^6^ cells of iPSC-derived intestinal organoids and 0.6 × 10^6^ iPSC-derived stromal cells growing on a porous membrane in an insert) and the neuronal model on a cell culture insert (total of 1 × 10^6^ cells of iPSC-derived neurospheres). Details on the differentiation and preculture of the iPSC-derived organ models can be found in the supplements. A pumping frequency of 0.5 Hz in the surrogate blood circuit and 0.4 Hz in the excretory circuit were set to ensure pulsatile fluid flow. The pressure was set to 450 mbar and the vacuum was set to -350 mbar for both pumps. The following medium was used in the four-organ-chip: Williams E without phenol red with/without glucose (PAN) with the addition of 1 mM nonessential amino acids, 2 mM L-glutamine, with/without 5% human AB serum, with/without ITS (human recombinant insulin: 0.01 g/l, human recombinant transferrin: 0.0055 g/l, selenious acid: 6.7 μg/l), 20 μg/ml gentamycin sulfate and 1 μg/ml amphotericin B. No human AB serum or ITS was added to the medium in the excretory circuit. A glucose feeding solution was prepared by dilution of a glucose-rich Nutriflex^®^ peri solution (B. Braun, Hessen, Germany) to a glucose concentration of 20 g/l. The pH of the glucose feeding solution was adjusted to 7.2. On day 0, the chip contained the following media: 225 μl intestinal medium on the apical side of the intestine model mixed with 50 μl glucose feeding solution, 1.37 ml four-organ-chip medium with glucose, human AB serum and ITS in the surrogate blood circuit, 75 μl neural cultivation medium on the apical side of the brain equivalent and 575 μl four-organ-chip medium with glucose but without human AB serum and ITS in the excretory circuit. The composition of the intestinal and neural cultivation medium are indicated in the supplements. Samples were taken from both medium reservoirs (100 μl) and from the apical side of the intestine equivalent (50 μl) daily. The medium removed was replaced with equal volumes of glucose-free medium for the surrogate blood medium reservoir and glucose-, serum- and ITS-free medium for the excretory medium reservoir. At the intestinal compartment, 50 μl glucose feeding solution was applied. In this way, 1 mg of glucose was fed daily to the system only through the intestine equivalent. Sets of chips (seven replicates) were cultivated for 7 and 14 days. Cellular samples were taken for immunohistological examinations (four models of one chip), qPCR (4 models per chip of 6 chips) and RNA sequencing (4 models of one chip) at the endpoints. Supernatants of ten chips were analyzed daily for their glucose and LDH content. Three independent four-organ-chip experiment were realized, whereupon the results are only shown for the described experiment.

**Figure 1.**
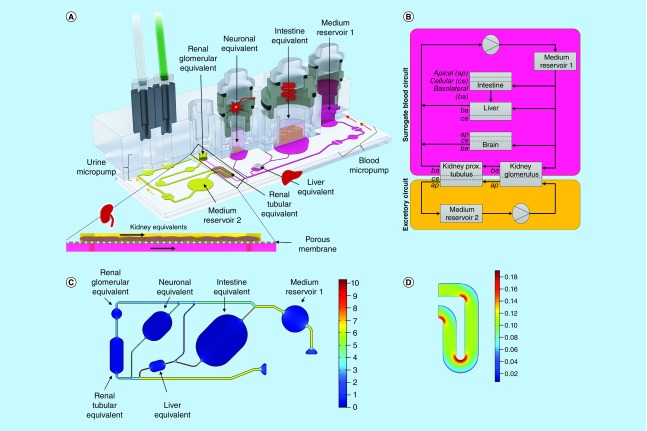
The microfluidic four-organ-chip at a glance. 3D view of the four-organ-chip **(A)**, physiologically inspired model of the four-organ-chip **(B)**. Pink: surrogate blood circuit, yellow: excretory circuit. Calculated velocity distribution (mm/s) at a flow rate of 16.9 μl/min in the surrogate blood circuit **(C)**. Distribution of the wall shear stress (Pa) in the tubular compartment of the excretory circuit at a mean shear stress of 0.1 Pa **(D)**.

Tissue viability was monitored daily by the measurement of LDH released in the supernatants of the three media pools: above intestine equivalent, surrogate blood medium reservoir 1 and excretory circuit medium reservoir 2 (Figure 1A). The LDH activity of the medium was measured using the Cytotoxicity Detection KitPLUS (Roche, Mannheim, Germany), according to the manufacturer's instructions with minor modifications. A standard curve based on the LDH Positive Control (L-LDH Standard stock solution from rabbit muscle, #10127230001, Sigma-Aldrich) was prepared. In brief, an amount of 12.5 μl of reagent was mixed with 12.5 μl of the sample. In case LDH concentration were outside of the measurement range, samples were diluted with 1% BSA in PBS and remeasured. Samples were incubated in 384-well microtiter plates at room temperature for 20 min on an orbital shaker. Absorbance readings at 490 nm with 680 nm background correction were performed in a microplate reader (ClarioStar, BMG Labtech, Ortenberg, Germany).

The metabolic activity of the tissues was monitored by daily measurement of glucose concentration in all three collected media supernatants. Samples were diluted 1:4 with distilled water and measured with the Glucose Hexokinase kit (REF 981779, Thermo Fisher Scientific, MA, USA) in the Indiko™ Clinical and Specialty Chemistry System (Thermo Fisher Scientific). Chip medium without glucose was used as a negative control and glucose feeding solution was used as a positive control of glucose measurement.

## Results

### Chip design

The four-organ-chip was designed to host cell culture spaces for intestinal organoids, liver spheroids, renal organoids and neurospheres in an arrangement mimicking the *in vivo* situation (Figure 1A). This combination of organs was chosen to allow for studies evaluating compound ADME (absorption, distribution, metabolism and excretion) profiles. Neuronal tissue was included as a fourth effector organ. Dimensional data of channels, cell culture compartments and flow characteristics were modelled to mirror the physiological relationship between them.

It was shown previously that renal cell differentiation and function was enhanced by the application of shear stress [16]. A separate circuit for renal cell culture was established to allow for adjustable shear rates on both sides of the kidney's tubule. Hence, the layout comprised two circuits (termed ‘surrogate blood circuit’ and ‘excretory circuit’) which overlapped in the kidney compartments and were separated by a porous cell culture-treated polycarbonate membrane (Figure 1A). One medium reservoir compartment in each circuit allowed sampling of supernatants. The medium was perfused through the microfluidic network by two incorporated, pneumatic micropumps – one for each circuit. The pumping technology was based on TissUse’ original MOC platform [17]. Long-term stability and adjustability to various flow rates had been validated previously [13,14,18].

A parallel, physiological-inspired flow scheme through the compartments and a medium flow partitioning mimicking physiological ratios of blood flow were adapted for quantitative *in vitro* to *in vivo* extrapolation (Figure 1B). Therefore, the dimensions of the channels of the blood circuit were based on scaled human physiology. Data for this were obtained from 1000 simulated male Europeans aged 40–60 and was gratefully provided by Certara/SimCyp. Average values for the four target organs are given in (Supplementary Table 1). Based on these values, organs received a distinct percentage of the blood flow from the main channel (Figure 1C). Modelling of the channel's flow resistances was carried out with MATLAB (Mathworks) and COMSOL Multiphysics 5.2a. Consequently, the flow was simplified to be laminar and steady. Hydrostatic pressures and any interaction of the two circuits were neglected.

The scaling of the organ compartments was based on the number of smallest functional units of the respective organs [19]. Ten liver lobuli – corresponding to 1 × 10^6^ cells – were placed in a compartment of 14.7 μl. Transferring this downscaling to the intestinal and neuronal equivalent results in a small intestinal and neuronal surface area of 12–24 mm^2^ and 4–6 mm^2^. Here, commercially available 24- and 96-well cell culture inserts were used to facilitate tissue model generation and integration. Therefore, the surface areas of the intestinal and neuronal equivalent were slightly above the calculated optimal downscaling areas – being 33 and 14 mm^2^, respectively.

Both circuits contained two functionally separated compartments at overlapping positions: One for the cultivation of podocytes – to emulate the renal glomeruli – and one for the cultivation of epithelial cells of the renal proximal tubule. This segregation of the kidney's functional units was adapted to account for substance filtration and reabsorption into the blood circuit. In contrast to the liver, the volume or number of cells does not define the renal functional output but rather the surface area that they provide for filtration and reabsorption. The complete surface of the human proximal tubule of a pair of kidneys is estimated to be 40–80 m^2^ when considering the epithelial cell's microvilli surface [20]. Scaling the kidney in accordance to the ten liver lobuli, the surface of the chip's tubules compartment had to remain between 6.7 and 26.7 mm^2^. We chose a surface of 13 mm^2^ considering the general size constraints of the chip. A single human glomerulus has a filtration surface of 0.17–0.21 mm^2^ [21,22]. Both human kidneys contain together approximately 1.5–2 million glomeruli [23]. By applying the same scaling factor, the glomerular compartment should have a surface of 2.6–4.2 mm^2^ in the four-organ-chip. We chose an area of 4 mm^2^ for the chip design. The output of the chip's micropump was adjusted to create an average shear stress of 0.1 Pa (1 dyn/cm^2^) on the surface of the epithelial cells of the proximal tubules (Figure 1D) [24].

### Human iPSC differentiations

All differentiated tissues used for the four-organ-chip coculture were derived from the same iPSC line, StemUse101/TISSUi001-A, to ensure an autologous culture of different tissues (Supplementary Figure 1). Liver, intestinal and neuronal tissue models were established as single static cultures before insertion into the four-organ-chip. Similarly, renal organoids were produced in static culture, but cells were dissociated on day 12 of differentiation and transferred into the renal circuit of each chip 6 days prior to the start of the coculture experiment (Supplementary Figure 1). We used standing 24-well cell culture inserts for the intestinal barrier model to obtain a permeable barrier separating the apical feeding space and the blood perfusion circuit in the four-organ-chip. An iPSC-derived stromal cell bed was combined here with preformed intestinal organoids. Neurosphere differentiation was performed in a bioreactor system (DasBox, Eppendorf, Hamburg, Germany) over 32 days, as previously described [25].

### Establishing the autologous four-organ-chip coculture

**Figure 2.**
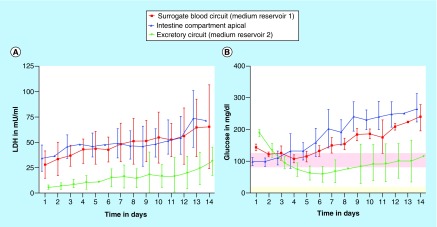
Systemic tissue viability in the four-organ-chip over 14 days of coculture. Measured by LDH activity **(A)** and glucose balance **(B).** Means with standard deviation are plotted. n = 10. The physiological glucose concentration range in the human blood [38] (red area) and urine [39] (light yellow area) of healthy people is drawn for comparison.

All tissue models were established in their specific iPSC culture media with tissue-specific growth factors or small molecules prior to their insertion into the four-organ-chip. During coculture, a common circulating medium supporting all tissues was mandatory to allow for cross-talk between organoids via cytokines and other molecular communication modes. Therefore, a common, growth factor-depleted medium was used in the surrogate blood circuit during coculture. The excretory circuit contained a similar medium which was additionally devoid of human AB serum and ITS. Neither media contained glucose, which was supplied exclusively to the apical side of the intestinal tissue model. All four organ models were sufficiently supplied with glucose due to the absorption through the intestinal model and subsequent circulation of the medium in both circuits (Figure 2B). The medium was perfused through the microfluidic network by two integrated pneumatic micropumps – one for each circuit – providing physiological pulsatile fluid flow at a microliter scale. The main flow rate in the blood circuit was 16.9 ± 0.7 μl/min analyzed by micro particle image velocimetry (μPIV – see Supplementary Material). This led to an approximate medium turnover time of 1.35 h. The excretory circuit had a flow rate of 6.6 ± 0.3 μl/min and a turnover time of 1.45 h. The minute amounts of enriched cultivation medium within the two circuits enabled cross-talk between the tissues. The μPIV analysis revealed correct perfusion of the intestinal and neuronal equivalents according to the criteria desired.

**Figure 3.**
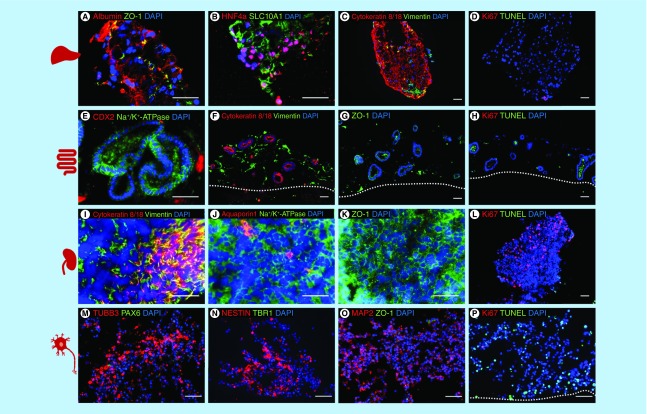
Characterization of the induced pluripotent stem cell-derived liver, intestinal, renal and neuronal model cocultivated in the four-organ-chip for 14 days. Immunostaining **A–P**: **A–D** liver spheroids: **(A)** albumin and ZO-1, **(B)** Hepatocyte nuclear factor 4 alpha and SLC10A1, **(C)** cytokeratin 8/18 and vimentin, **(D)** Ki67 and TUNEL. **(E–H)** intestinal model: **(E)** CDX2 and Na^+^/K^+^-ATPase, **(F)** cytokeratin 8/18 and vimentin, **(G)** ZO-1, **H:** Ki67 and TUNEL. **(I–L)** renal model: **(I)** cytokeratin 8/18 and vimentin, **(J)** aquaporin 1 and Na^+^/K^+^-ATPase, **(K)** ZO-1, **(L)** Ki67 and TUNEL. **(M–P)** neuronal model; **(M)** TUBB3 and PAX6, **(N)** nestin and TBR1, **(O)** MAP2 and ZO-1, **(P)** Ki67 and TUNEL. Scale 50 μm.

### Establishing the iPSC-derived organ model coculture in the four-organ-chip

Coculture viability and performance was assessed daily by analyzing LDH and glucose in the culture medium (Figure 2) and by microscopic inspection. The latter was feasible through the chip's transparent bottom allowing live tissue imaging. Overall cell turnover was studied by measurement of LDH concentration in all compartments (Figure 2A). LDH concentrations were higher in the surrogate blood circuit and the apical intestinal compartment compared with the excretory circuit. Together with the significant glucose difference between the circuits, this indicates that the kidney membrane represents a diffusion barrier, retarding the exchange of small molecules such as glucose as well as proteins such as LDH (140 kDa).

Similarly, glucose concentrations were measured to determine whether all tissues were supplied sufficiently. Feeding glucose only apically through the intestinal equivalent was shown to be sufficient to supply both the surrogated blood circuit and excretory circuit over 14 days of chip coculture (Figure 2B). Glucose values of the surrogated blood circuit were in the range of physiological glucose fasting concentration of 81–126 mg/dl [26] until day 5, but exceeded these concentration for the following days. Glucose values in the excretory circuit exceed the physiological range of 2–20 mg/dl [27] at all time points, due to the permeability of the kidney membrane. Nevertheless, a significant glucose gradient between the surrogated blood and the excretory urine circuit was established from day 5 on.

All organ equivalents were assessed on the protein expression level on day 14 of coculture to determine their identity and viability (Figure 3). Staining for apoptotic cells (TUNEL) and proliferating cells (Ki67) showed a high viability in the liver, intestinal and renal model on day 14 of coculture; only the neuronal model showed some dead cells (Figure 3D, H, L & P).

The liver spheroids fused during the coculture in the chip and showed expression of albumin, the tight junction protein ZO-1, the hepatic transcription factor HNF4 alpha and SLC10A1, a liver bile acid transporter (Figure 3A & B). The parenchymal marker cytokeratin 8/18 was expressed in the cytoskeleton of the cells and the mesenchymal marker vimentin showed the distribution of the mesenchymal stromal cells inside the liver spheroids (Figure 3C).

Intestinal organoids had a lumen and expressed the Na^+^/K^+^-ATPase transporter. Few cells within the organoids were positive for the intestinal transcription factor CDX2 (Figure 3E). Cytokeratin 8/18 was only expressed in the epithelial cells of the intestinal organoids and vimentin only in mesenchymal cells in the stromal bed around the organoids (Figure 3F). The tight junction protein ZO-1 was also expressed in the cell membranes of the intestinal organoids as well as in the stromal cells (Figure 3G).

As expected, kidney organoids showed a mixed culture of epithelial cells (cytokeratin 8/18) and mesenchymal cells (vimentin) (Figure 3I); the transporter Na^+^/K^+^-ATPase was expressed selectively in the cell membranes (Figure 3J) and the proximal tubules marker aquaporin 1 was only expressed in a few cells (Figure 3J). The tight junction protein ZO 1 was expressed in the cell membranes of the renal organoids (Figure 3K). Additionally, we saw many proliferating cells in the kidney organoids and only a few apoptotic cells (Figure 3L).

The neurospheres self-assembled inside the Transwell systems into larger structures during the 14 days of four-organ-chip cultivation. Tissues appeared to be heterogeneous with distinct TUBB3, MAP2 and nestin positive areas (Figure 3M–O), which indicates structural changes of the neurospheres induced by the four-organ-chip coculture. No ZO1 positive lumen surrounded by PAX6 positive cells were found after 14 days.

**Figure 4.**
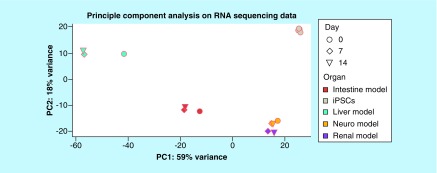
Principle component analysis on RNA sequencing data of the liver, intestine, brain and kidney model cultivated over 0, 7 and 14 days in the four-organ-chip. Five different human iPSC lines were analyzed for comparison. iPSC: Induced pluripotent stem cell.

RNA sequencing and the following principle component analysis on all four different iPSC-derived models cocultivated over 14-days in the four-organ-chip, and five different iPSC lines were analyzed regarding the first two principal components (Figure 4). Distinct clustering of each model was visible. Interestingly, the kidney and brain model clustered close to each other. The shift from day 0 to day 7 and 14 was striking for the liver and intestine model. Coculture in the four-organ-chip under dynamic conditions was, therefore, having a profound effect on the RNA expression profile. This was also confirmed by the high-throughput barrier multiplex qPCR analysis of the intestinal and kidney models (Supplementary Figure 2). Investigation of 94 different barrier genes showed 49 significantly different regulated genes (Supplementary Figure 2A). Distinct clustering of the intestinal versus the renal models was also obvious in the principal component analysis (Supplementary Figure 2B).

**Figure 5.**
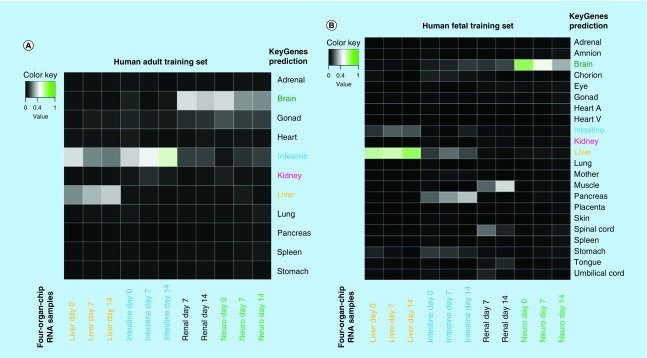
KeyGenes prediction on RNA sequencing data from the four-organ-chip experiment. KeyGenes prediction with the human adult training set **(A)** and the human fetal training set **(B)**. The identity scores range from 0 (black – no match) to 1 (green – high match). The rows represent the 11 **(A)** or 22 **(B)** organs from the human training set and the columns depict the four-organ-chip cocultured RNA samples. Light grey and green boxes show the predicted tissues for the given samples by KeyGenes.

The RNA sequencing data from the four-organ-chip experiments were used for comparative analysis using KeyGenes. It is an algorithm to predict the identity and to determine the identity scores of queried samples to provided transcriptional profiles of different organs or cell types [28,29]. The intestine, liver and brain model were identified by KeyGenes using the human adult training set (Figure 5A & B). The intestine model cocultivated over 14 days in the four-organ-chip showed the highest identity score with the intestine when using the adult training set (Figure 5A). Only the kidney model was not predicted appropriately; instead, it was predicted as a brain sample. This is probably due to an incomplete renal differentiation and a coexisting differentiation into neuronal cells in addition to the renal cells. The liver spheroids showed a higher identity score when the human fetal training set was used instead of the human adult training set (Figure 5B). Therefore, we assume that the liver spheroids have a fetal phenotype. Moreover, the neuronal model also had a higher identity score in the fetal training set. Principle component analysis (Figure 4) and KeyGenes prediction from RNA sequencing data (Figure 5) showed that the intestinal and liver models not only maintained their phenotype in the common media, but also even differentiated further along their destined paths.

## Discussion & conclusion

This study presents a successful long-term cocultivation of four different iPSC-derived tissues from a single donor cultured in a physiologically inspired microfluidic device. The morphology and differentiation status of the tissue models after 14 days of coculture was comparable to the results published in the protocols which were used for the iPSC differentiation [30–34], although no tissue-specific growth factors were added to the medium in the four-organ-chip. The basic medium with 5% human AB serum was sufficient to sustain all four human iPSC-derived tissues for 14 days. There was no dedifferentiation observed for the liver and intestinal models; we could even remark an advanced maturation of these tissues over time.

Still, the tissue maturation is yet not fully attained and calls for further developments considering scaffolds and coculture with stabilizing and supporting cells. The state-of-the-art iPSC differentiation protocols mostly lack the generation of fully matured organ equivalents and rather produce fetal tissue models. We aspire toward a closed, iPSC-derived endothelialization of the channels and tissues in the chips. This will support the use of full blood for nutrition and oxygen supply enhancing physiological relevance and therewith eventually tissue differentiation.

KeyGenes prediction presents that the iPSC-derived liver spheroids show more similarities with a fetal than an adult liver (Figure 5A & B). We are confident that the development of improved iPSC differentiation protocols regarding growth factors and scaffolds will lead to more mature cells and tissues. Regarding the intestinal model, the highest consent of the KeyGenes prediction can be seen between the 14-day intestinal cocultures model and the adult intestine (Figure 5A). The kidney model was the only tissue that was not successfully predicted by KeyGenes. Instead, KeyGenes predicted a brain or muscle organ from the adult or fetal training set, respectively (Figure 5A & B). We assume that the rare renal organoids, which arouse during differentiation, were overgrown by coexisting cells due to high proliferation in the organoids. Therefore, the differentiation efficiency into purer renal organoids is to be optimized. The glomerulus is designed to house filtering renal cells, such as podocytes, on one side and endothelial cells on the other. Furthermore, the tubular loop should receive proximal tubular epithelial cells, which reabsorb solutes and water back into the blood circuit, which is also ideally lined by endothelial cells. Although the chip design and the organoids on top of the glomerulus support the tightness of the porous polycarbonate membrane, the biological barrier function of the intestine equivalent and the renal equivalent was incomplete in this study, as there was no tight layer of endothelial or epithelial cells holding the barrier. Nevertheless, the technical barrier between the excretory circuit and the blood surrogate circuit could maintain discrete glucose and LDH concentrations in the two circuits. The excretory circuit contained much less LDH than the surrogated blood circuit (Figure 2). However, in future experiments the barrier function needs to be improved and validated for example with glucose transport blockers.

No further maturation of the neuronal tissue was observed, which is supported by the absence of the cortical development marker TBR1 after 14 days of chip coculture. This on the other hand could also be explained by the generally late appearance of this marker around day 50 of neural spheroid cultivation [35]. We believe that in future the introduction of blood–brain barrier specific endothelial cells [36] will provide an active transport of nutrients into the neuronal compartment and thereby enable a further maturation or at least an equilibrium of the neuronal model. Furthermore, daily media exchanges with glucose-free maintenance medium to remove waste products or the culture under air-liquid interface as shown by Tieng and colleagues [37] to increase the oxygen supply of the tissue might improve the culture conditions of the neuronal model. KeyGenes prediction showed the close similarity of the initial neuronal culture to fetal tissue (Figure 5B), however, there is a decrease over time, which together with the declining MAP2 expression supports the assumption that culture conditions are not yet ideal.

Overall, the combination of human autologous organ models and their systemic communication in a dynamic MPS circulation are envisioned lead to a significant improvement in physiological relevance of model systems and, eventually, in the prediction of drug response. Primary cells still have a higher maturation status in comparison to the state of the art iPSC-derived cells. But obtaining different tissue from the same donor in quantities and qualities sufficient for an industrial testing of substances is not feasible. The generation of high numbers of standardized iPSC-derived tissues will eliminate the bottleneck of primary cells. Furthermore, repeated tests on cells of the same origin are possible by using iPSC-derived material.

## Conclusion & future perspective

The assembly of tissues from one single population of iPSCs will ultimately enable the creation of a donor-on-a-chip for drug screening. Receiving tissues from a single donor will be even more important once immune components are present in the chip cocultures. Hence, rejection reactions as a result of the systemic connection of tissues with different genetic background can be prevented. Furthermore, the generation of a patient-on-a-chip from a disease donor gives great potential regarding personalized medicine and allows, for instance, the focus on genetic mutations of a single donor. In the future, the chips need to be handled by an automatization platform which includes the transfer of the organ models into the chips, cultivation of the chips, the medium supply, medium sampling and microscopic analysis for tissue quality assurance. This will lead to increased reproducibility and decreased variability of the chip assays at large throughput for reasonable costs. The patient-on-a-chip holds great promise for further investigations, especially in the emerging field of individualized cellular immunotherapies in oncology, autoimmunity and transplantation medicine.

**Executive summary**

- Four-organ-chip interconnecting miniaturized human intestine, liver, brain and kidney equivalents.
- All four organ models were predifferentiated from induced pluripotent stem cells from the same healthy donor and integrated into the microphysiological system.
- Coculture of the four autologous tissue models in one common medium deprived of tissue specific growth factors was successful over 14-days.
- No added growth factors present in the coculture medium.
- Intestine, liver and neuronal model maintained defined marker expression.
- Renal model was overgrown by coexisting cells and did not further differentiate.
