# Supplementary material for: Autologous induced pluripotent stem cell-derived four-organ-chip
Source: Future Sci OA. 2019 Sep 10;5(8):FSO413. doi: 10.2144/fsoa-2019-0065 (PMC6745596; doi:10.2144/fsoa-2019-0065)
Supplement: Supplementary file 2 [file fsoa-05-413-s2.pdf]

Paper supplements: Autologous iPSC-derived four-organ-chip

Supplementary materials and methods

iPSC generation and cultivation

Cell culture plates and components were purchased from Corning U.S. and cultures were incubated at 37 °C and 5 % CO_2, unless otherwise stated. The human iPSC line StemUse101 (TissUse GmbH) was derived from peripheral blood mononuclear cells from a 52-year-old male donor. Human blood samples were donated with informed consent and ethics approval (Ethic Committee Berlin Chamber of Physicians, Germany) in compliance with the relevant laws. Reprogramming was performed by Phenocell SAS with an episomal vector (Epi5 Kit, Thermo Fisher Scientific A15960). The iPSCs were maintained in feeder-free conditions in StemMACS iPS-Brew XF (Miltenyi) on growth factor-reduced (GFR) Matrigel® (1:100 diluted in KO/DMEM F12; Thermo Fisher Scientific) on dishes treated for cell culture. The iPSCs were passaged every five to seven days using Accutase®, 4,000 – 6,000 cells/cm² were seeded and medium was added with 10 µM Rock Inhibitor Y-27632 (Cayman). StemMACS iPS-Brew XF medium without Rock Inhibitor was renewed after 48 h, following a daily medium exchange. The iPSC differentiations into stromal cells, hepatocyte-like cells, intestinal organoids, renal cells and cortical neurospheres were performed with iPSCs in passage 18 to 25.

Differentiation into iPSC-derived stromal cells

Stromal cell differentiation was carried out with modifications from Zou et al. [1]. The iPSCs were grown on GFR Matrigel until 80 % confluent. The medium was changed stepwise to stromal cell medium (50 % DMEM HG with 50 % Ham's F-12, 10 % FCS, 15 mM HEPES and 1 % penicillin-streptomycin: P/S) with one transitional day of 50:50 stromal cell medium with StemMACS iPS-Brew XF. The medium was changed daily during the first four days and, subsequently, renewed every two or three days. After three weeks, cells were split 1:3 with trypsin on 0.1 % gelatin (Sigma)-coated cell culture dishes; 10 µM Rock Inhibitor Y-27632 was added to the medium only for the split. Cells were split every two weeks or when confluent. The iPSC-derived stromal cells in passage four were used for the intestine and liver spheroids.

Differentiation into iPSC-derived hepatocyte-like cells and liver spheroids formation

Hepatocyte-like cell differentiation was carried out with modifications from Szkolnicka et al. [2]. The iPSCs were differentiated into definitive endoderm (DE) with STEMdiff™ Definitive Endoderm Kit (TeSR™-E8™ Optimized) (StemCell), according to the manufacturer's instructions with minor modifications. The iPSCs were split with Accutase and seeded with 33,000 cells/cm² on GFR Matrigel (1:100 diluted in KO/DMEM F12) in StemMACS iPS-Brew XF supplemented with 10 µM Rock Inhibitor and STEMdiff™ Definitive Endoderm TeSR™-E8™ Supplement (1:20). Supplement A and B treatment was performed two days after seeding. After 24 h, only supplement B was added for three days, as described in the instructions. The cell layer was then treated for five days with 1 % dimethyl sulfoxide in KO/DMEM (Thermo Fisher Scientific), 20 % KnockOut™ serum replacement (KSR) (Thermo Fisher Scientific), 1 mM glutamine, 1 % nonessential amino acids, 0.1 mM 2-mercaptoethanol (Thermo Fisher Scientific) and 1 % P/S. Due to pronounced cell death during the first two days of the dimethyl sulfoxide treatment, the cells were gently washed with PBS before the medium was replaced. Afterwards, the cell layer was treated for an additional five days with HZM medium (HepatoZYME-SFM (Thermo Fisher Scientific) medium with 1 mM nonessential amino acids, 2 mM L-glutamine, 2 % KSR, 10 ng/mL human HGF (Miltenyi), 10 ng/mL human FGF-4 (Peprotech), 10 ng/mL human oncostatin M (Miltenyi), 0.1 µM dexamethason (Ehrenstorfer GmbH) and 1 % P/S). Gentle medium exchange was performed every other day. Liver spheroids were formed by combining iPSC-derived hepatocyte-like cells and iPSC-derived stromal cells using Corning® 384-well spheroid microplate. The iPSC-derived hepatocyte-like cells and iPSC-derived stromal cells were detached with trypsin, centrifuged and diluted in HZM medium (described previously) supplemented with 10 µM Rock Inhibitor and 5 % bovine serum albumin fraction V, fatty acid free (BSA-FAF) (SERVA). A 50 µL cell suspension containing 4.8×10^4 hepatocyte-like cells and 0.2×10^4 iPSC-derived stromal cells was pipetted into each access hole with a 96-well pipette (Platemaster®, Gilson). After two days of culture on an orbital shaker (Corning), the spheroids were transferred with wide-bore tips to ultra-low attachment 24-well plates. Twenty spheroids were collected together to form a single liver spheroids in the respective culture compartment of the four-organ-chip.

Differentiation into iPSC-derived intestinal organoids and cell culture insert® seeding

Intestinal organoid differentiation was carried out with modifications from Kauffman et al.[3] and McCracken et al [4]. The iPSCs were differentiated into DE with STEMdiff™ Definitive Endoderm Kit (TeSR™-E8™ Optimized), as described above. Following the DE stage, the cell layer was treated with hindgut medium (Advanced DMEM/F-12; Thermo Fisher Scientific) with 50 ng/mL KGF (Peprotech), 0.1 % BSA-FAF, 15 mM HEPES, 1x B27-Supplement (Thermo Fisher Scientific) and 1 % P/S for four days. The hindgut medium was added with 50 ng/mL KGF, 2 % BSA-FAF, 15 mM HEPES, 1x B27-Supplement, 1 % P/S, 1x insulin-transferrin-selenium (ITS), 200 mg/L, ethanolamin (Sigma) and 2 µM retinoic acid (Alfa Aesar) for the following three days. Subsequently, the 3D cell layer was scraped off with a pipette tip and small pieces were transferred with a cooled wide-bore pipette tip into cool 100 % Matrigel (354234). The suspension was mixed gently without producing bubbles and 50 µL Matrigel cell suspension was transferred immediately with a cooled wide-bore pipette tip into the middle of a 37 °C pre-warmed 24-well plate. The Matrigel was solidified in the incubator for 10 min, then 500 µL intestinal medium (Advanced DMEM/F-12 with 2 mM L-glutamine, 15 mM HEPES, 1x B27-supplement, 1 % P/S, 500 ng/mL R-Spondin-1 (Peprotech), 100 ng/mL Noggin (Peprotech) and 100 ng/mL EGF (StemCell) was added. Intestinal organoids were passaged every two weeks by resuspending the organoids 10x with a 200 µL pipette in PBS, centrifuging at 300 *g* at 4 °C for 3 min and transferring the organoids in Matrigel, as described above. Expansion of organoids was increased stepwise from 1:2 to 1:20. The intestinal medium was changed every two to three days. For the intestinal model cell culture insert (Millicell PCF 0.4 µm pores PIHP01250) 0.6 x 10^6 iPSC-derived stromal cells per cell culture insert were seeded in stromal cell medium. After two days, the cell culture inserts were washed gently with PBS, 50 µL cooled Matrigel was added and the intestinal organoids in passage six were seeded on top of the stromal cells in 70 µL Matrigel-medium (50/50) suspension. One Matrigel organoid 24-well droplet was used for two cell culture inserts. After the Matrigel solidified, 100 µL intestinal medium was added into the cell culture insert and 500 µL underneath. Intestinal medium was changed every two to three days. The intestinal models on cell culture inserts were cultivated for two weeks before transferring them into the four-organ-chip for further co-culture.

Differentiation into iPSC-derived renal cells

Renal cell differentiation was carried out with modifications from Morizane and Bonventre [5]. The iPSCs were grown until 50 % confluent. Subsequently, the cells were washed gently with PBS and the medium was replaced by advanced RPMI 1640 (Thermo Fisher Scientific) with 2 mM L-glutamine, 5 µg/mL gentamycin sulfate and 0.25 µg/mL amphotericin B (renal basal medium). 8 µM CHIR99021 (LC Labs) was added to the medium during the first four days. Medium was replaced every other day. Afterwards, the renal basal medium was added with 10 ng/mL Activin A (Peprotech) for three days without medium exchange. The cells were then washed with PBS and 10 ng/mL FGF-9 (Peprotech) was added to the renal basal medium. Two days later, an induction with 3 µM CHIR99021 and 10 ng/mL FGF-9 was carried out. Subsequently, the cells were detached using Accutase and about 10^6 cells were injected gently into the excretory circuit of each four-organ-chip. The excretory circuit was coated previously with 1 % GFR Matrigel and filled with renal basal medium supplemented with 10 ng/mL FGF-9. Two days later, 50 % renal basal medium supplemented with 10 ng/mL FGF-9 was replaced in the chip. Renal cells were injected six days before the start of the four-organ-chip co-culture.

Differentiation into iPSC-derived cortical neurospheres and Transwell® seeding

Differentiation of iPSCs into cortical progenitor cell spheroids, referred to as neurospheres, was performed in a DASbox® Mini Bioreactor System (Eppendorf) [6]. The culture protocol was adapted from Rigamonti et al. [7]. Initial spheroid formation of iPSCs in the DASbox bioreactor was performed as described by Abecasis et al. [8]. The iPSCs were inoculated as a single cell suspension with a concentration of 2.5 x 10^5 cells/mL in 125 mL StemMACS iPS-Brew XF supplemented with 10 µM Rock Inhibitor Y-27632 in a 250-mL fermentation vessel (DASbox Mini Bioreactor System). The vessel was equipped with a trapezoid-shaped impeller, a submerged tube for media withdrawal, a non-submerged tube for media feeding, and a dissolved oxygen and pH sensor. Sensors and pumps were calibrated according to the manufacturer's protocols. The submerged sampling tube was equipped with a 10 µm porous filter to allow the washout of single cells while retaining the iPSC spheroids in the vessel. The vessel was kept at a temperature of 37 °C and aerated with 3 sl/hour (5 % CO_2, variable O_2) and a stirrer speed of 80 rpm. The aeration with oxygen was adjusted automatically by DASware control software (Eppendorf) to achieve a stable oxygen saturation at 19 % dissolved oxygen. No medium was

exchanged for the first 24 h after inoculation. Starting at day two, medium was exchanged via the tubing system at a rate of 120 mL/day at day two, 60 mL/day at day three, 90 mL/day at day four and 120 mL/day at day five. After five days of cultivation, the process was stopped temporarily, and the spheroids were used for cortical differentiation. Agitation was stopped and the spheroids were allowed to settle at the bottom of the vessel. The culture medium was removed completely and the spheroids suspended in a defined volume of fresh iPSC culture medium. The suspension was mixed well and a sample was taken for cell counting with an automated cell counter (Nucleocounter NC-200, Chemometec). Cells were reintroduced as spheroids into the DASbox bioreactor system with 7.5×10^5 cells/mL in 100 mL StemMACS iPS-Brew XF. The system was operated with the same process parameters, as described for the spheroid formation. After 24 h (day two of differentiation), the medium was completely exchanged manually with KSR medium (KO-DMEM (Thermo Fisher Scientific) with 15 % KSR, 1 % nonessential amino acids, 2 mM L-Glutamine, 1 % P/S and 50 µM 2-mercaptoethanol). The medium was constantly exchanged at a rate of 2.08 mL/h from day two on. Only KSR medium was fed until day 4. From then on to day 10, the KSR medium was gradually diluted with neural induction medium (DMEM/F12 (Thermo Fisher Scientific) with 1 % N2 (Thermo Fisher Scientific), 2 % B27 without vitamin A (Thermo Fisher Scientific), 1 % nonessential amino acids, 2 mM L-Glutamine and 1 % P/S. Only neural induction medium was fed from day 10 to day 30. The cell culture medium was supplemented with 10 µM SB431542 (Miltenyi) and 1 µM LDN193189 (Sellekchem) from day one to day seven. From day two to four, medium was furthermore supplemented with 2 µM XAV939 (Cayman). After 30 days, neurospheres were collected and cell debris was removed with a 37 µm reversible strainer (Stemcell). The spheroids were resuspended in a defined volume of neural cultivation medium (Neurobasal (Thermo Fisher Scientific) with 1 % N2, 2% B27 without vitamin A, 1% nonessential amino acids, 2 mM L-glutamine and 1 % P/S. The suspension was mixed well and a sample was taken for cell counting. The neurospheres were further cultivated in neural cultivation medium in an Erlenmeyer flask on an orbital shaker. The next day, the neurospheres were transferred into a 96-well Transwell® system with a 1 µm pore polyester membrane (Corning). A total of 2×10^6 cells was transferred into each Transwell. Spheroids were cultured for 24 h in neural cultivation medium before individual Transwells were transferred into the microfluidic chip system.

Analysis

Characterization of fluid dynamics in the four-organ-chip

The two integrated peristaltic micropumps generated the flow in both microfluidic circuits. Their working principle is explained elsewhere [9]. The deflection of the pumps' membranes was controlled by an external unit (TissUse GmbH) that managed the frequency of deflection, pressure and vacuum. Their interplay defined the flow rate within the two circuits. Micro particle image velocimetry (µPIV) was applied to determine the flow rate for the set process parameters. Red blood cells were utilized to visualize the flow. Therefore, red blood cells were suspended in PBS at a hematocrit of 2.5 %. The µPIV analyses were carried out in straight channels. Throughout the measurements, the chip was equipped with the same cell culture inserts and volumes as in the corresponding biological experiments. The flow was recorded with a high-speed CMOS camera (HXC40, Baumer Optronic) coupled to an inverted microscope (Axiovert, Zeiss). Magnification was set to either 2.5 or 5x, which resulted in a resolution of 0.23 and 0.46 px/µm, respectively. The acquisition rate's upper limit was 2,583 fps. The displacement of the red blood cells in recordings of up to 7.7 s was computed by the open source toolbox "PIVlab" [10,11]. Only velocity vectors in the central portion of the channels were regarded to determine the flow rate of the laminar plug flow. Details of the underlying equations have been discussed elsewhere [9]. The flow rates were averaged from triplicate measurements. The standard deviation is given.

Immunohistochemistry

Immunohistochemical analyses were performed by staining with the following primary antibodies: hepatocyte nuclear factor 4 (alpha R&D, mouse anti-human), CDX2 (BioGenex, mouse anti-human), Cytokeratin 8/18 (Santa Cruz Biotechnology Inc., mouse anti-human), Ki-67 (eBioscience, mouse anti-human), TUBB3 or beta-3 tubulin (eBioscience, mouse anti-human), MAP2 (Merck Millipore, mouse anti-human), Na^+/K^+-ATPase (abcam, rabbit anti-human), vimentin (Thermo Fisher Scientific, rabbit anti-human), SLC10A1/NTCP (abcam, rabbit anti-human), PAX6 (BioLegend, rabbit anti-human), aquaporin1 (abcam, mouse anti-human), ZO-1 (Proteintech, rabbit anti-human), albumin (sigma, mouse anti-human), TBR1 (abcam, rabbit anti-human) and nestin (eBioscience, mouse anti-human). All antibodies and the used concentrations are listed in table S3 and S4. The Apo-Direct Apoptosis Detection

Kit (Thermo Fisher Scientific) was used for the detection of apoptotic cells, according to the manufacturer's instructions. Briefly, representative central 7 µm cryosections of the tissues were fixed in acetone at −20 °C for 10 min and washed twice with PBS. Tissue sections were then incubated with the respective primary antibody in 10 % goat or donkey serum in PBS for 2 h and washed afterwards twice with PBS. Afterwards sections were incubated with CF488A goat anti-rabbit IgG and CF594 goat anti-mouse or CF488A donkey anti-mouse and CF594 donkey anti-goat (all purchased from Biotium) together with 1 µg/mL DAPI (Sigma-Aldrich) for 45 min at room temperature for visualization. Images were obtained using a Keyence BZ-X700E fluorescent microscope.

RNA sequencing

A total of 16 RNA samples were analyzed with the NextSeq 500 sequencer (Illumina) to study RNA expression. The Illumina TruSeq stranded total RNA protocol was used including rRNA depletion (Cat. No. RS-122-2201). Cluster generation and sequencing was carried out by using the Illumina NextSeq 500 system with a read length of 75 nucleotides, according to the manufacturer's guidelines. Sequence reads that passed the Illumina quality filtering were considered for alignment. Raw FASTQ files were checked for quality, aligned with the STAR mapper [Reference PMID 23104886] (2.5.3a) to the human genome assembly version GRCh37 and transcripts were quantified using Salmon [Reference PMID 28263959] (0.9.1). The resulting quast files were subjected to DESeq2 [Reference PMID 25516281] (1.18.1) analysis using the R statistical language. Samples were normalized and the dispersions were estimated using the default DESeq2 settings. DESeq2 was then used to identify differentially expressed genes for each pairwise comparison between organ and iPSCs. KeyGenes (www.keygenes.nl) [12] was used to predict the identity of the four-organ-chip co-cultivated iPSC-derived samples. Annotated count values for each gene and sample are provided in an extra excel file in the supplements.

Barrier genes high-throughput multiplex qPCR

A total of 250 ng RNA per sample were transcribed to cDNA using the High-Capacity cDNA Reverse Transcription Kit (Applied Biosystems, Cat. No. 4368814), according to the manufacturer's protocol. Ninety-four targets were investigated: PPIA, 18SrRNA, b-Actin, GAPDH, B2M, Claudin-1, Claudin-2, Claudin-3, Claudin-4, Claudin-5, Claudin-6, Claudin-7, Claudin-8, Claudin-9, Claudin-10a, Claudin-10b,

Claudin-11, Claudin-12 tv1, Claudin-12 tv2, Claudin-12 tv3, Claudin-14, Claudin-15, Claudin-16, Claudin-17, Claudin-18 tv1b, Claudin-18 tv2a, Claudin-19, Claudin-20, Claudin-22, Claudin-23, Claudin-24, Claudin-25, ZO-1, ZO-2, ZO-3, Jam-1, Jam-2, Jam-3, Occludin, Tricellulin, ABCB1, ABCC1, ABCC2, ABCC3, ABCC4, ABCC5, ABCG2, CAT1, CAT3, ENT1, LAT1, MCT1, MCT8, SLC1A1, SLC2A1, Insulin receptor, Transferrin receptor, LRP1, LRP8, E-cadherin, CDH5, VEGF, b-catenin, Vimentin, Fibronectin, Filagrin, E-selectin, vWF, Cytokeratin-1, Cytokeratin-4, Cytokeratin-5, Cytokeratin-8, Cytokeratin-10, Cytokeratin-13 tv1, Cytokeratin-13 tv2, Cytokeratin-14, Cytokeratin-16, Cytokeratin-18, Cytokeratin-19, Aquaporin-1, Aquaporin-3, Aquaporin-4, Aquaporin-7, Aquaporin-10, Aquaporin-11, Mucin-1A, Mucin-1B, Mucin-2, Mucin-3A, Mucin-4, Mucin-5AC, Mucin-13, Mucin-18 and Mucin-20. They were preamplified using tenfold concentrated primer pools mixing with Qiagen Mastermix applying the following program: 15 min at 95 °C for HotStar Plus Taq Polymerase (Qiagen, Cat. No. 203603), 18 cycles (with 40 s at 95 °C, 40 s at 60 °C, 80 s at 72 °C) and 7 min at 72 °C. High-throughput qPCR was accomplished with a Biomark™ System containing an IFC Controller HX and 96.96 Dynamic Arrays™ IFC, according to the manufacturer's instructions. In brief, 96 sample wells were loaded with DNA Mix containing Tagman GeneExpression Mastermix, DNA binding dye sample loading reagent, EvaGreen binding dye and 1:8 diluted preamplified cDNA, whereas 96 target wells were filled with the Assay mix containing Assay loading reagent and according primers. After qPCR and data allocation, the Ct values of the targets were normalized to endogenous control PPIA. The ΔCt values were used for the following statistical analysis (PCA, Heatmaps) applying the software Qlucore Omics Explorer 3.3.

Supplementary Figures

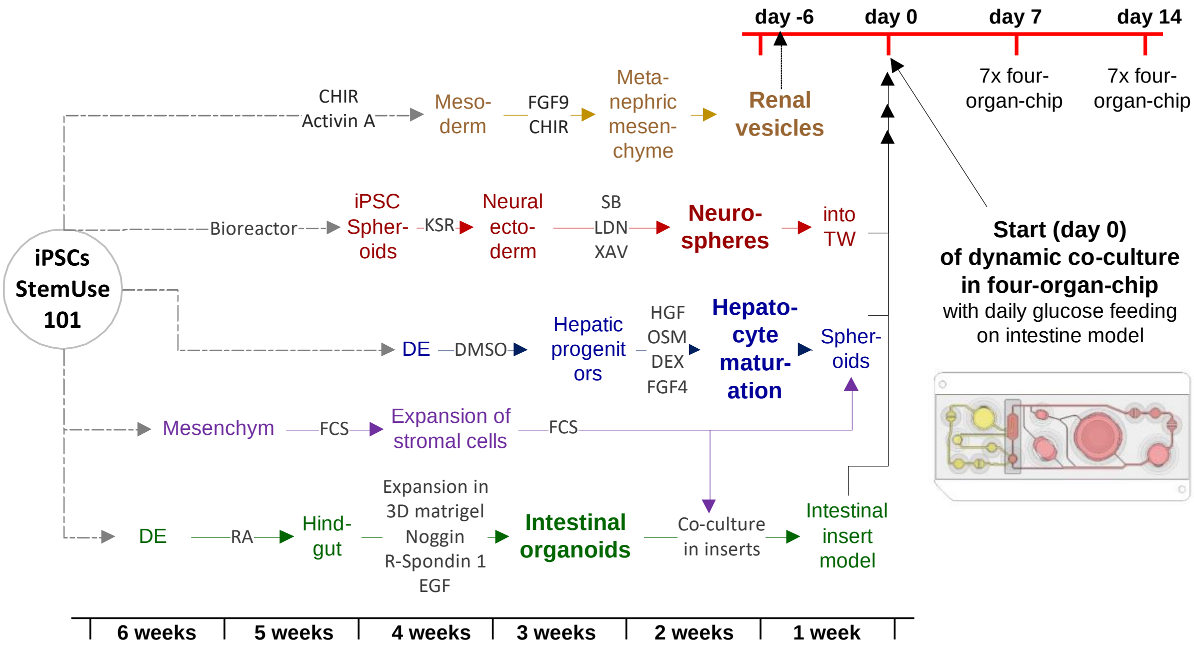

Figure S 1: Schematic time frame of the iPSC differentiation into kidney organoids, liver spheroids, neurospheres and intestinal organoids, transfer and co-cultivation in four-organ-chip. DE: definitive endoderm.

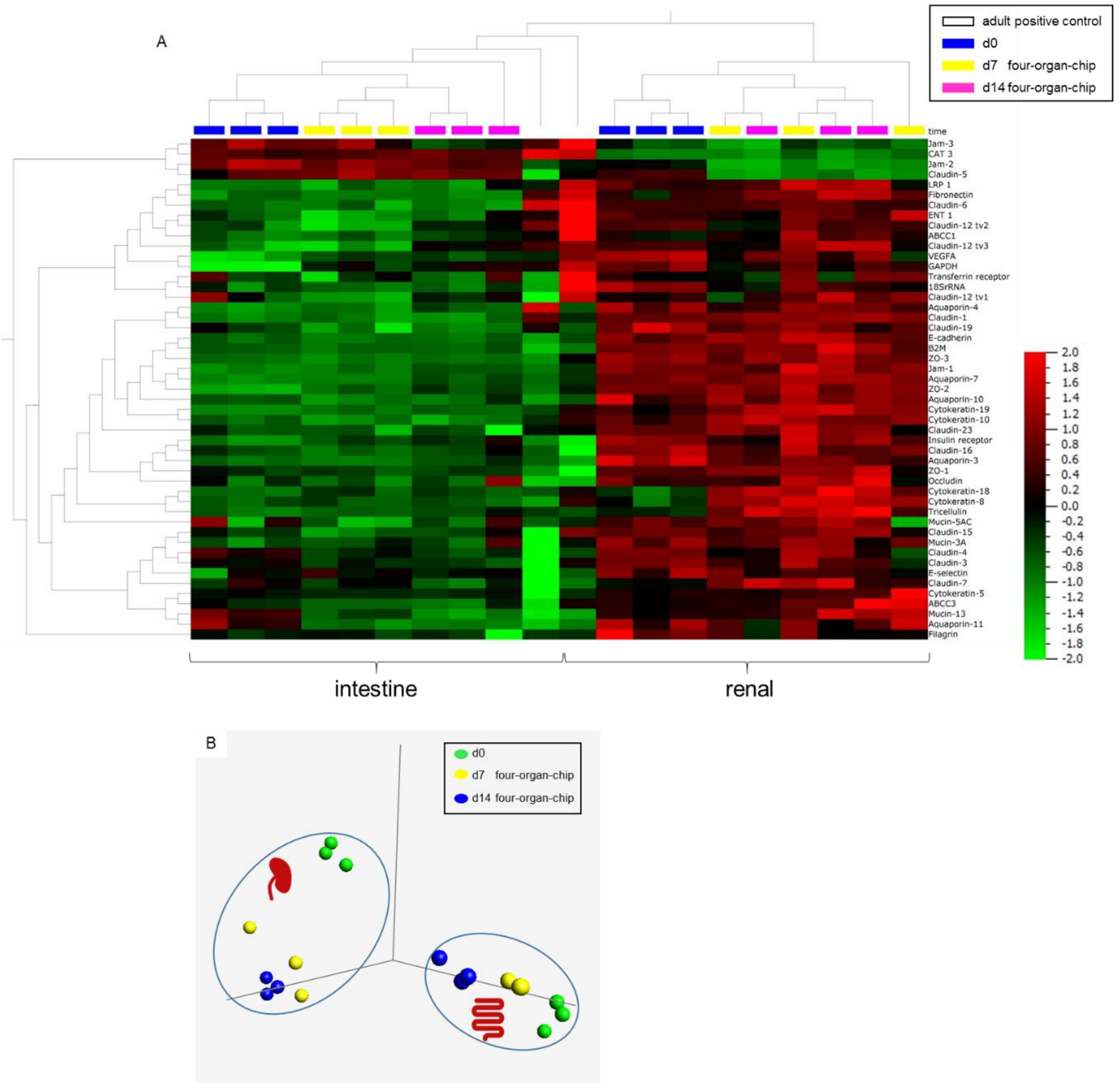

Figure S 2: Heatmap analyses of the high-throughput multiplex barrier qPCR data of the intestinal and kidney model on day 0 (blue), day 7 (yellow) and day 14 (pink) of co-culture in the four-organ-chip. An intestinal and renal adult positive sample was measured as a comparison (white). Forty-nine of 94 significant different target genes are shown. A multigroup comparison at p = 0.05 was performed. Three replicates per time point (A). Principle component analysis of the high-throughput multiplex barrier qPCR data of the intestinal and kidney model cultivated for 14 days in the four-organ-chip (B).

Supplementary Table

Table S 1: Blood flow through key organs averaged from simulated data of 1,000 male Europeans. The ratios were calculated disregarding the organs not modeled in the chip. Only bold figures are totaled. The blood flow through the hepatic portal vein was simplified to be equal to the blood flow through the small intestine.

Organ	Blood flow [L/h]	Ratio of blood flow [%]
Small intestine	**64.3**	**35.3**
Liver	82.0	45.2
through portal vein	~64.3	35.3
through hepatic artery	**~17.7**	**9.9**
Kidney	**61.1**	**33.7**
Brain	**38.6**	**21.1**
Total (arterial blood)	**181.7**	**100**

Table S 2: Volume of chip compartments.

Organ compartment	Volume [µl]
Intestine apical	275
Surrogate blood circuit	1370
Neural compartment apical	75
Excretory circuit	575
**Total**	**2295**

Table S 3: Primary human antibodies for immunohistochemical staining.

Primary antibody	Host	Number	Manufacturer	Concentration
Albumin	Mouse	A6684	Sigma	1:100
Aquaporin 1	Mouse	ab9566	Abcam	1:100
CDX2	Mouse	MU392A-100	BioGenex	1:100
Cytokeratin 8/18	Mouse	sc-70939	Santa Cruz	1:100
HNF4α	Mouse	MAB4605-SP	R&D	1:100
Ki67	Mouse	14-5699-82	eBioscience	1:100
Na^+/K^+-ATPase	Rabbit	ab76020	Abcam	1:100
Nestin	Mouse	14-9843-82	eBioscience	1:100
PAX6	Rabbit	901301	Biolegend	1:200
SLC10A1/NTCP	Rabbit	ab131084	Abcam	1:100
TBR1	Rabbit	ab31940	Abcam	1:100
TUBB3 or β 3 Tubulin	Mouse	14-4510-82	eBioscience	1:100
Vimentin	Rabbit	PA5-27231	Thermo	1:100
ZO-1	Rabbit	21773-1-AP	Proteintech	1:100

Table S 4: Secondary antibodies for immunohistochemical staining.

Secondary antibody	Host	Number	Manufacturer	Concentration
Donkey anti-mouse CF488A	Donkey	20014	Biotium	1:200
Donkey anti-goat CF594	Donkey	20116	Biotium	1:200
Goat anti-mouse CF594	Goat	20119	Biotium	1:200
Goat anti-rabbit CF488A	Goat	20012	Biotium	1:200

References

1. Zou L, Luo Y, Chen M, *et al.* A simple method for deriving functional MSCs and applied for osteogenesis in 3D scaffolds. *Sci. Rep.* 3(1), 2243 (2013).

2. Szkolnicka D, Farnworth SL, Lucendo-Villarin B, Hay DC. Deriving functional hepatocytes from pluripotent stem cells. *Curr. Protoc. Stem Cell Biol.* 2014(August), 1g.5.1-1g.5.12 (2014).

3. Kauffman AL, Ekert JE, Gyurdieva A V, Rycyzyn MA, Hornby PJ. Directed differentiation protocols for successful human intestinal organoids derived from multiple induced pluripotent stem cell lines. *Stem Cell Biol. Res.* [Internet]. 2(1), 1 (2015). Available from: http://www.hoajonline.com/stemcells/2054-717X/2/1.

4. Mccracken KW, Howell JC, Wells JM, Spence JR. Generating human intestinal tissue from pluripotent stem cells in vitro. *Nat. Protoc.* 6(12), 1920–1928 (2011).

5. Morizane R, Bonventre J V. Generation of nephron progenitor cells and kidney organoids from human pluripotent stem cells. *Nat. Protoc.* 12(1), 195–207 (2016).

6. Koenig L, Ramme A, Faust D, Marx U. Scalable production of human pluripotent stem cell-derived neurospheres in a suspension bioreactor system. 49(0), 5130264.

7. Rigamonti A, Repetti GG, Sun C, *et al.* Large-scale production of mature neurons from human pluripotent stem cells in a three-dimensional suspension culture system. *Stem Cell Reports* [Internet]. 6(6), 993–1008 (2016). Available from: http://dx.doi.org/10.1016/j.stemcr.2016.05.010.

8. Abecasis B, Aguiar T, Arnault É, *et al.* Expansion of 3D human induced pluripotent stem cell aggregates in bioreactors: Bioprocess intensification and scaling-up approaches. *J. Biotechnol.* [Internet]. 246, 81–93 (2017). Available from: http://dx.doi.org/10.1016/j.jbiotec.2017.01.004.

9. Schimek K, Busek M, Brincker S, *et al.* Integrating biological vasculature into a multi-organ-chip microsystem. *Lab Chip* [Internet]. 13(18), 3588 (2013). Available from: http://xlink.rsc.org/?DOI=c3lc50217a.

10. Thielicke W. The flapping flight of birds: Analysis and application. Anal. Appl. (2014).

11. Thielicke W, Stamhuis EJ. PIVlab – Towards User-friendly, Affordable and Accurate Digital Particle Image Velocimetry in MATLAB. *J. Open Res. Softw.* [Internet]. 2(1), e30 (2014). Available from: http://openresearchsoftware.metajnl.com/articles/10.5334/jors.bl/.

12. Roost MS, Van Iperen L, Ariyurek Y, *et al.* KeyGenes, a tool to probe tissue differentiation using a human fetal transcriptional atlas. *Stem Cell Reports*. 4(6), 1112–1124 (2015).
